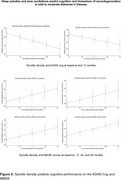# Sleep spindles and slow oscillations predict amyloid beta, tau pathology, and cognitive decline in persons with mild to moderate Alzheimer's Disease.”

**DOI:** 10.1002/alz70856_104172

**Published:** 2025-12-25

**Authors:** Arsenio Paez, Sam O Gillman, Shahla Bakian Dogaheh, Anna Carnes, Farida Dakterzada, Ferran Barbe, Thien Thanh Dang‐Vu, Gerard Piñol‐Ripoll

**Affiliations:** ^1^ Concordia University, Montreal, QC, Canada; ^2^ University of Oxford, Oxford, Oxfordshire, United Kingdom; ^3^ Centre de Recherche de l’Institut Universitaire de Gériatrie de Montréal, Montreal, QC, Canada; ^4^ Hospital Universitari Santa Maria de Lleida, IRBLleida, Lleida, Spain; ^5^ Hospital Universitari Santa Maria LLeida, LLeida, Spain; ^6^ Centre de recherche de l'Institut universitaire de gériatrie de Montréal, Montreal, QC, Canada; ^7^ Institut de Recerca Biomédica de Lleida (IRBLLeida), Lleida, Spain; ^8^ Cognitive Disorder Unit, Hospital Universitari Santa Maria, Lleida, Spain

## Abstract

**Background:**

Sleep plays vital roles in brain‐health and cognition, including regulating clearance of β‐amyloid (Aβ) and tau proteins that hallmark Alzheimer's disease (AD). Changes in sleep physiology can predate cognitive symptoms by decades in AD, but it remains unclear which sleep characteristics predict cognitive and neurodegenerative changes after AD onset.

**Methods:**

Using data from a prospective cohort study of mild‐to‐moderate AD (*n* = 60, 30 female, mean age 74.7) in Lleida, Spain, we analysed non‐rapid eye‐movement sleep spindles and slow oscillations (SO) at baseline and their associations with baseline amyloid‐beta and tau, and with cognition from baseline to three‐years follow‐up.

Participants underwent polysomnography (PSG), blood and cerebrospinal fluid draws for amyloid and tau at baseline, and neuropsychological assessment at baseline, 12, 24 and 36 months with the Mini‐Mental Status Examination (MMSE), California verbal learning test (CVLT), Rey‐Osterrieth Complex Figure Test (ROCF), Alzheimer's Disease Assessment Scale–Cognitive Subscale (ADAS‐Cog).

Spindle and SO detection were performed using in‐house, open‐source software packages developed at Concordia University. Associations between SO and spindle and SO duration, density, power, amplitude, AD biomarkers, and cognition from baseline to 36 months were investigated with false discovery rate‐adjusted generalised linear models controlling for age, sex, apnoea‐hypopnea index.

**Results:**

We found previously unreported associations between spindle and SO activity, biomarkers, and cognition in persons with AD. Higher spindle and SO density, duration, amplitude and power predicted significant changes in amyloid‐beta 42 (β= 69.3 *p* =  0.001), phosphorylated (pTau‐181) (β= 7.92, *p* = 0.001) and total‐tau, and tau/Aβ42 (β= ‐0.52, *p* = 0.001), clinically and statistically significantly lower Alzheimer's Disease Assessment Scale Cognitive Subscale (better cognitive performance) (β= ‐9.0, *p* = 0.001) and higher Mini‐Mental State Examination scores (better cognitive performance) (β= 15.2, *p* = 0.01) from baseline to 36‐months, and significant changes in verbal and visual memory on the ROCF and CVLT. Spindles and SO activity also mediated the effect of pTau181/aβ42 on cognition (41‐81%), while pTau181/aβ42 moderated the effect of spindles and SO on cognition.

**Conclusions:**

Our findings demonstrate that spindle and SO activity during sleep constitute predictive and non‐invasive biomarkers of neurodegeneration and cognition in AD patients and novel treatment targets for delaying cognitive decline and AD progression.